# Sodium New Houttuyfonate Induces Apoptosis of Breast Cancer Cells via ROS/PDK1/AKT/GSK3β Axis

**DOI:** 10.3390/cancers15051614

**Published:** 2023-03-06

**Authors:** Lixin He, Huili Feng, Baoyi Yin, Wenxuan Li, Xiao Wang, Talha Umar, Hongbo Gao, Ning Zhou, Changwei Qiu

**Affiliations:** 1 Department of Clinical Veterinary Medicine, College of Veterinary Medicine, Huazhong Agricultural University, Wuhan 430070, China; 2 Department of Animal Physiology and Molecular Biology, College of Animal Husbandry Engineering, Henan Vocational College of Agriculture, Zhengzhou 451450, China

**Keywords:** breast cancer, sodium new houttuyfonate, ROS, apoptosis

## Abstract

**Simple Summary:**

Breast cancer treatment has long been a problem plaguing women’s health. In order to explore whether sodium new houttuyfonate (SNH) has a potential therapeutic effect on breast cancer, this study jointly demonstrated that SNH has a significant apoptotic effect on breast cancer through biogenic analysis and research trials. This effect is achieved by promoting the excessive accumulation of reactive oxygen species (ROS), inducing mitochondrial damage, regulating the aggregation of apoptotic proteins near mitochondria, and targeting the PDK1-AKT-GSK3β pathway. This study demonstrated the potential therapeutic effect of SNH and provided a reference for the application of SNH in breast cancer.

**Abstract:**

Background: Sodium new houttuyfonate (SNH) has been reported to have anti-inflammatory, anti-fungal, and anti-cancer effects. However, few studies have investigated the effect of SNH on breast cancer. The aim of this study was to investigate whether SNH has therapeutic potential for targeting breast cancer. Methods: Immunohistochemistry and Western blot analysis were used to examine the expression of proteins, flow cytometry was used to detect cell apoptosis and ROS levels, and transmission electron microscopy was used to observe mitochondria. Results: Differentially expressed genes (DEGs) between breast cancer-related gene expression profiles (GSE139038 and GSE109169) from GEO DataSets were mainly involved in the immune signaling pathway and the apoptotic signaling pathway. According to in vitro experiments, SNH significantly inhibited the proliferation, migration, and invasiveness of MCF-7 (human cells) and CMT-1211 (canine cells) and promoted apoptosis. To explore the reason for the above cellular changes, it was found that SNH induced the excessive production of ROS, resulting in mitochondrial impairment, and then promoted apoptosis by inhibiting the activation of the PDK1-AKT-GSK3β pathway. Tumor growth, as well as lung and liver metastases, were suppressed under SNH treatment in a mouse breast tumor model. Conclusions: SNH significantly inhibited the proliferation and invasiveness of breast cancer cells and may have significant therapeutic potential in breast cancer.

## 1. Introduction

Breast cancer is a focus of global attention because of its high incidence and high mortality rates. According to the 2020 report from the International Agency for Research on Cancer (IRAC), breast cancer has surpassed lung cancer as the most common cancer worldwide, with an incidence rate of 11.7% [1]. Breast cancer has great clinical heterogeneity, and patients with different subtypes of the disease have varying clinical prognoses. Canine mammary tumors are frequently regarded as models for the study of human breast cancer because of their similarities in pathology, histology, and shape [2]. Additionally, phosphatidylinositol-4,5-bisphosphate 3-kinase (PIK3CA) mutations, abnormalities in the PI3K-AKT pathway, and a crucial gene implicated in cancer initiation and development are shared between human breast cancer and canine mammary tumors [3,4].

Currently, many medications used to treat breast cancer in clinical treatment have varying degrees of severe side effects [5]. Recently, bioactive compounds extracted from traditional Chinese medicine have shown potential anti-cancer abilities. Sodium new houttuyfonate (SNH) is the chemical synthesis of houttuynia, and it has anti-inflammatory [6], anti-fungal [7,8] and anti-cancer properties [9,10]. Studies have suggested that SNH can exert anti-inflammatory effects by promoting the generation of reactive oxygen species (ROS) [6], which are considered as a product of oxygen consumption and cell metabolism. Endogenous ROS include superoxide anions, hydrogen peroxide, and hydroxyl radicals [11,12]. Excessive ROS may disrupt mitochondrial functions, mainly manifesting as mitochondrial membrane potential decrease, mitochondrial transcription factor A decrease, mitochondrial mass increase, and mitochondrial DNA fragmentation increase [13,14]. In general, endogenous ROS are maintained within a normal range. ROS levels outside the normal range will lead to the occurrence of disease [15]. Therefore, ROS can be considered as a potential target for cancer therapy.

Studies have shown that abnormal activation of the PI3K-AKT pathway plays a critical role in the occurrence, metastasis, and drug resistance of breast cancer, and it is closely related to the prognosis of breast cancer, all of which make it a potential therapeutic target for breast cancer [16,17]. The biological function of the PI3K-AKT pathway can be modulated by ROS. The combination of polydatin and 2-deoxy-d-glucose promotes apoptosis in breast cancer by eliminating endogenous ROS production [11], as does hyperoside [18]. Moreover, total secondary saponin from the rhizome of Anemone raddeana showed anti-proliferation and pro-apoptotic activities on MCF-7 cells through the ROS-mediated mitochondrial apoptosis pathway [19]. Genistein can lead to mitochondrial dysfunction through ROS accumulation, induce the inactivation of PI3K-AKT, and synergistically promote the anti-tumor effect of Centchroman [20]. GSK3β is a downstream target of AKT. The serine 9 phosphorylation of GSK3β is negatively correlated with the activity of GSK3β, which is adversely associated with the viability of breast cancer cells [21].

Thus, this study investigated whether SNH could induce mitochondrial dysfunction through ROS overgeneration and inactive the PDK1-AKT pathway to induce apoptosis of breast cancer cells in vitro and in vivo.

## 2. Materials and Methods

### 2.1. Bioinformatics Analysis

Two expression profiles related to breast cancer (GSE139038 and GSE109169) were screened from GEO DateSets (https://www.ncbi.nlm.nih.gov/gds/?term=, accessed on 15 August 2022. The differentially expressed genes (DEGs) and the common DEGs shared between the two datasets were shown using a Venn diagram (http://bioinformatics.psb.ugent.be/webtools/Venn/, accessed on 15 August 2022). The Gene Ontology resource (GO, http://geneontology.org/, accessed on 16 September 2022) and the Kyoto Encyclopedia of Genes and Genomes (KEGG, https://www.kegg.jp/, accessed on 16 September 2022) were used to analyze the enrichment of GO and KEGG involved in these common DEGs, and bubble charts of GO and KEGG enrichment analysis were plotted using bioinformatics (https://www.bioinformatics.com.cn, accessed on 16 September 2022), an online platform for data analysis and visualization. An interaction network of these common DEGs was constructed, and the protein interaction network of some important genes was emphatically analyzed using Cytoscape software. 

The molecular structure of SNH was assessed using PubChem (https://pubchem.ncbi.nlm.nih.gov/, accessed on 16 August 2022). The potential target genes of SNH were predicted through SwissTargetPrediction (http://www.swisstargetprediction.ch/, accessed on 16 August 2022). The expression analysis and prognosis analysis of target genes were conducted individually using GEPIA (http://gepia.cancer-pku.cn/detail.php, accessed on 17 September 2022) and Kaplan–Meier Plotter (https://kmplot.com/analysis/index.php?p=service, accessed on 17 September 2022).

### 2.2. Reagents and Antibodies

The reagents used were as follows: sodium new houttuyfonate (Yuanye, Shanghai, China, CAS: 112714-99-5); docetaxel (Yuanye, Shanghai, China); hydroxypropyl-β-cyclodextrin (HP-β-CD; Solarbio, Beijing, China); N-acetyl-cysteine (NAC; Macklin, Shanghai, China); matrix adhesive (Biozellen, Frontier, NE, USA); Opti-MEM I medium (Gibco, Billings, MA, USA); and crystal violet (BioSharp, Hefei, China).

The kits used were as follows: Cell Counting Kit-8 (Hycezmbio, Wuhan, China), ROS Detection Kit (Hycezmbio, Wuhan, China), BCA Protein Quantification Kit (Hycezmbio, Wuhan, China), Apoptosis Detection Kit (Hycezmbio, Wuhan, China); and Transwell chamber (Corning, NY, USA).

According to the protocol recommended by the manufacturer, the following antibodies were used for Western blot or immunofluorescence: Anti-BAX (Wanleibio, WL01637), Anti-p-GSK3β (Wanleibio, WL03683), Anti-β-actin (ABclonal, AC038), Anti-Bcl-2 (ABclonal, A19693), Anti-cleaved PARP p25 (ABclonal, A19612), Anti-caspase-9 (ABclonal, A0281), Anti-PDK1 (ABclonal, A0834), Anti-p-PDK1 (ABclonal, AP0426), Anti-AKT (ABclonal, A20799), Anti-p-AKT (ABclonal, WLP001), Anti-GSK3β (ABclonal, A11731), Anti-MMP1 (ABclonal, A22080), HRP Goat Anti-Rabbit IgG (ABclonal, AS014), Alexa Flour 594-Goat Anti-Rabbit IgG (ABbox, AD9279), and Cy3 Goat Anti-Rabbit IgG (H + L) (ABclonal, AS007).

### 2.3. Cell Culture

Human breast cancer cell line MCF-7 (kindly donated by Zhiqiang Dong Laboratory at Huazhong Agricultural University) and canine mammary cancer cell line CMT-1211 (kindly provided by the Degui Lin Laboratory at the China Agricultural University) were used in this study. Both cell lines were cultured in DMEM (Gibco) medium containing 10% fetal bovine serum (Hycezmbio, Wuhan, China) and 2% penicillin-streptomycin solution (Gibco) at 37 °C with 5% CO_2_. 

### 2.4. Cell Viability Assay

Cells at a density of 1 × 10^4^ cells/well were seeded into 96-well plates. When the density reached 50–60%, different concentrations of SNH were added. After treatment for 24 h, 10 µL (5 mg/mL)/well of Cell Counting Kit-8 (CCK-8, Hycezmbio, Wuhan, China) was added and incubated with cells at 37 °C for 30 min. Cell viability was measured through absorbance (optical density) with a microplate reader (Bio-Rad Instruments, Hercules, CA, USA) at 450 nm.

Another set of experiments was also conducted. The cells were cultured in 96-well plates with different concentrations of SNH for different time (0, 12, 24, 36, 48, and 60 h). The cell culture medium containing the drugs was changed once every 12 h. Other operations remained unchanged unless otherwise indicated.

### 2.5. Cell Migration Assay

Cell migration was detected using a wound-healing migration assay. The cells were inoculated into 6-well plates with 1 × 10^5^ cells/well. When the cells reached 80–85% confluence, a 200 μL plastic sucker scraped the cell layer once, and the exfoliated cells were washed with sterile phosphate buffered saline (PBS). The cells were cultured with serum-reduced Opti-MEM I medium (Gibco, Billings, MA, USA). The wounds were photographed when the scrape wound was introduced (0 h) and at a designated time (24 h) using an inverted microscope.

### 2.6. Cell Invasion Assay

This step was conducted according to the reagent instructions (Biozellen, Frontier, NE, USA). Matrigel A (2×) at a concentration of 1/15 was added into 8 μm transwell chambers. Then, the matrigel in chambers was refrigerated at 4 °C for 3 h, and 7.5 × 10^4^ cells/well which have been treated with SNH for 24 h were suspended in 100 µL culture solution and added to the upper compartment of the chamber. DMEM medium containing 10% fetal bovine serum was added to the lower compartment of the chamber. The cells were cultured in an incubator at 37 °C for 24 h. Next, the cells in the chambers were fixed with methanol and stained with crystal violet. The images were photographed using an optical microscope (Olympus, Tokyo, Japan).

### 2.7. Apoptosis Assay

Apoptosis was detected using Annexin V-FITC (fluorescein isothiocyanate) and PI (propidium iodide) double staining. The staining procedures and detection method were conducted according to the Apoptosis Detection Kit’s instructions (Hycezmbio, Wuhan, China). The cell density was adjusted to 1 × 10^6^/mL and suspended at 250 μL binding buffer. The cells were gently vortexed and incubated with 5 μL of Annexin V-FITC and 10 μL of PI at room temperature for 10 min against exposure to light. The apoptosis rates were detected using flow cytometry (CytoFLEX, Beckman, State Key Laboratory of Agricultural Microbiology at Huazhong Agricultural University).

### 2.8. Intracellular ROS Assay

ROS generation was detected using the fluorescent probe DCFH-DA. The cells were collected after being treated with drugs. The staining procedures and detection method for the cells were conducted according to the ROS Detection Kit’s instructions (Hycezmbio, Wuhan, China). The cell density was adjusted to 1 × 10^6^/mL. The cells were stained with 1:1000 diluted probe at 37 °C for 30 min against exposure to light and washed twice with PBS. The reactive oxygen positive control reagent was Rosup, provided by the ROS Detection Kit, with a concentration of 50 mg/mL. Rosup was diluted in serum-free DMEM medium at 1:1000 and incubated cells at room temperature for 30 min. The DCFH-DA probe was loaded in accordance with the above procedures. The ROS levels were detected using flow cytometry (CytoFLEX, Beckman, State Key Laboratory of Agricultural Microbiology at Huazhong Agricultural University).

### 2.9. Transmission Electron Microscopy

Cells were seeded into 6-well plates at a density of 1 × 10^5^ cells/well. When the confluence rate of the cells reached 60–70%, the cells were treated with varying concentrations of SNH for 24 h. Samples were processed successively: fixed, dehydrated, and permeated. Resin blocks containing samples were cut into ultrathin sections (80 nm thick) with a Leica UC6 ultrathin microtome. Ultrathin sections were observed using a transmission electron microscope (TEM) (H7650, Hitachi, Japan) at 100 kV after being stained with uranium acetate.

### 2.10. Western Blot Analysis

Protein immunoblotting was performed according to previous methods [22]. The total cellar proteins were harvested with RIPA containing 1% PMSF and 1% phosphatase inhibitors. The proteins’ concentrations for each treatment group were determined using a BCA Kit. Proteins were separated with electrophoresis using 10% sodium dodecyl sulfate (SDS)-polyacrylamide gel and then transferred to polyvinylidene fluoride (PVDF). The embranees were successively incubated with antibodies and secondary antibodies. Anti-β-actin was considered an internal reference protein. Protein expression was detected with the French Vilber Lourmat FX7 detection system. 

### 2.11. Cellular Immunofluorescence Staining

Cells were seeded into 24-well plates at a density of 1 × 10^4^ cells/well. SNH at different concentrations or other drugs were added and remained for 24 h. After treatment, the cells were fixed with 4% (*v*/*v*) paraformaldehyde for 30 min and permeated with 0.5% (*v*/*v*) Triton X-100 in PBS for 20 min. Subsequently, the cells were blocked with 5% (*v*/*v*) goat serum for 2 h and incubated with primary antibodies overnight at 4 °C. The cells were incubated with Alexa Flour 594-Goat Anti-Rabbit IgG or Cy3 Goat Anti-Rabbit IgG (H + L) in the dark for 1 h and stained with 4,6-diamidino-2-phenylindole (DAPI, Beyotime, Shanghai, China) for 10 min. In this experiment, the cells were washed three times with PBS between each treatment. Images were observed using a fluorescence microscope (Olympus, Tokyo, Japan).

### 2.12. In Vivo Experiment

Five week-old Balb/C female mice were purchased from the Experimental Animal Center of Huazhong Agricultural University (Wuhan, China) one week prior to the experiment. When the mice were six weeks old, 1 × 10^6^ cells/mouse were inoculated at the 4th accessory mammary pad. After 10 days of injection, they were randomly divided into 5 groups: blank control group, negative control group (HP-β-CD, 0.1 mL (30% *w*/*v*)/mouse, i.p.), docetaxel group (8 mg/kg, i.p.), low-dose SNH group (20 mg/kg, i.p.), high-dose SNH group (40 mg/kg, i.p.),and mock group, which were administered by intraperitoneal injection once every 2 days for 22 days. During the treatment, the mice were weighed, and the tumor volume was measured every 2 days. After that, the mice were euthanized according to the ethical requirements of experimental animals of Huazhong Agricultural University (HZAUMO-2022-0125) and the United States National Institutes of Health. Tumor tissues and lung, heart, liver, spleen, kidney, and other tissues of the mice were harvested and fixed in 4% paraformaldehyde. Tumor volume (V) = 0.5 × length × width^2^. 

### 2.13. Mouse Tissue Section Staining

Subsequently, the tumor and organs were dehydrated after being fixed in formaldehyde for 48 h and embedded in paraffin, which were cut into 5 µm thick sections for hematoxylin and eosin (H&E) staining. An optical microscope (Olympus, Tokyo, Japan) was used for image acquisition.

Tumor sections were also used for immunofluorescence staining. After being dewaxed to water with xylene and gradient ethanol, tumor sections were soaked in citric acid antigen repair buffer (pH 6.0) and heated in a microwave oven for antigen repair. The following procedures were similar to those of cellar immunofluorescence staining. Finally, the glass sheet needed to be sealed with an anti-fluorescent quenching agent. A fluorescence microscope (Olympus, Tokyo, Japan) was used for image collection.

### 2.14. Statistical Analysis

The dates were obtained as mean ± SD of at least three independent experiments. Differences between groups were calculated using one-way ANOVA or nonlinear regression (GraphPad Prism 8). A level of * *p* < 0.05 was considered significant, ** *p* < 0.01, *** *p* < 0.001, or **** *p* < 0.0001 were considered extremely significant, and *p* > 0.05 (ns) was considered not significant.

## 3. Results

### 3.1. DEGs’ Analysis of Breast Cancer Based on the GEO Database

**Figure 1:**
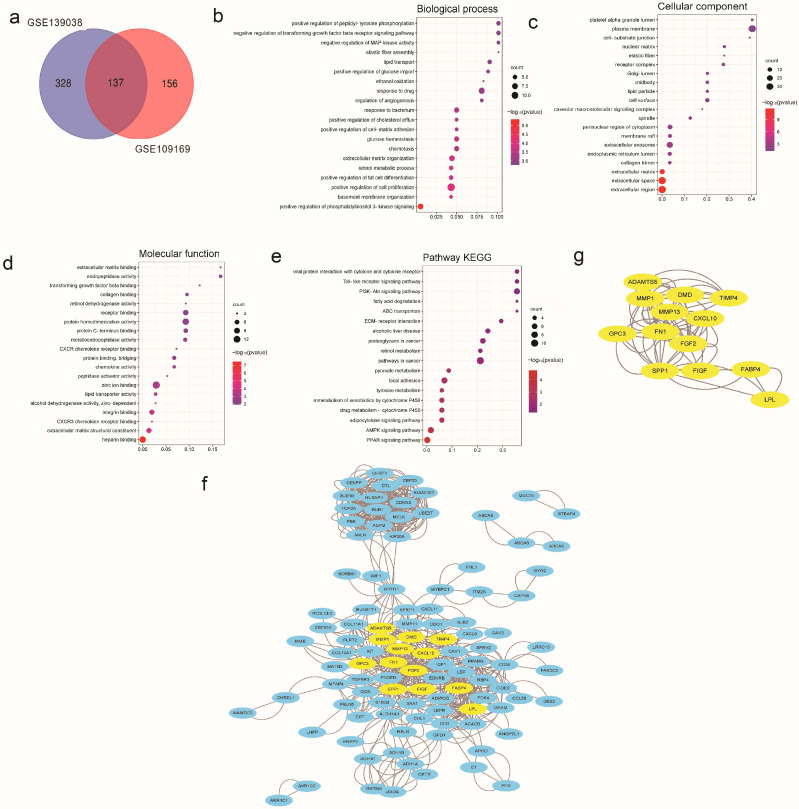
DEGs’ analysis associated with breast cancer in the GEO database. (**a**) The common DEGs between GSE139038 and GSE109169 are shown with a Venn diagram. (**b**–**e**) Bubble charts were used to show the GO and KEGG enrichment analyses for these DEGs. (**f**) Interaction network analysis of the common DEGs was performed using Cytoscape software. (**g**) Emphasizing the interaction network analysis of DEGs related to MMP1. All results are expressed as mean ± SD of three independent experiments.

Given that the occurrence and development of breast cancer involves expression changes of multiple genes, we analyzed the expression profiles related to breast cancer in the GEO database (GSE139038 and GSE109169). In these two GEO datasets, GEO2R was applied to analyze these DEGs [23,24]. The intersection of the two datasets was calculated by drawing Venn diagrams on the online tool, as shown in Figure 1a. The results show that there are 137 DEGs. These DEGs were further analyzed based on Gene Ontology (GO) annotations and KEGG pathways. We found that the 137 DEGs were involved in biological processes, including the regulation of PI3K signaling, cell proliferation, etc. (Figure 1b). The cellular components involved included the extracellular region, the extracellular matrix, etc. (Figure 1c). The molecular functions of these DEGs were heparin binding, integrin binding, CXCR3 chemokine receptor binding, etc. (Figure 1d). KEGG analysis showed that these DEGs were mainly involved in the PPAR signaling pathway, the PI3K-AKT signaling pathway, etc. (Figure 1e). 

To further study the interactions of these DEGs, we constructed an interaction network diagram of these DEGs using Cytoscape, highlighting the interaction relationships of some important DEGs, as shown in Figure 1f,g.

### 3.2. Network Pharmacological Analysis of SNH

**Figure 2:**
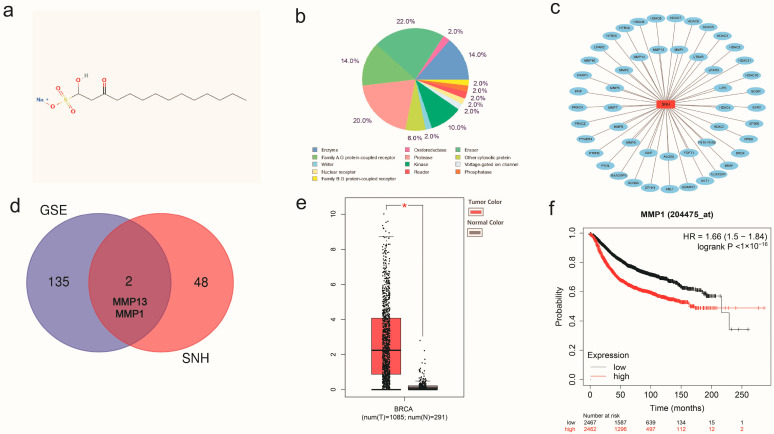
Network pharmacology analysis of SNH. (**a**) The chemical structural formula of SNH. (**b**) Category statistics of potential targets of SNH. (**c**) The potential targets of SNH were predicted using SwissTargetPrediction. (**d**) The common target genes of SNH and these two profiles (GSE139038 and GSE109169). (**e**) Differential expression of MMP1 in breast cancer. (**f**) Relationship between MMP1 and prognosis in breast cancer. All results are expressed as mean ± SD of three independent experiments. * *p* < 0.05.

First, we recognized the molecular structure of SNH in PubChem (Figure 2a) and predicted the potential target genes of SNH using SwissTargetPrediction (Figure 2b,c). After further analysis, we found that two potential target genes of SNH were consistent with 137 DEGs, namely MMP13 and MMP1 (Figure 2d). The expression of MMP1 between breast cancer tissues and normal tissues was significantly different. The invasiveness of breast cancer is tightly linked to MMP1, and high expression of MMP1 usually predicts a poor prognosis in breast cancer (Figure 2e,f).

### 3.3. SNH Inhibited the Proliferation and Invasiveness of Breast Cancer Cells

**Figure 3:**
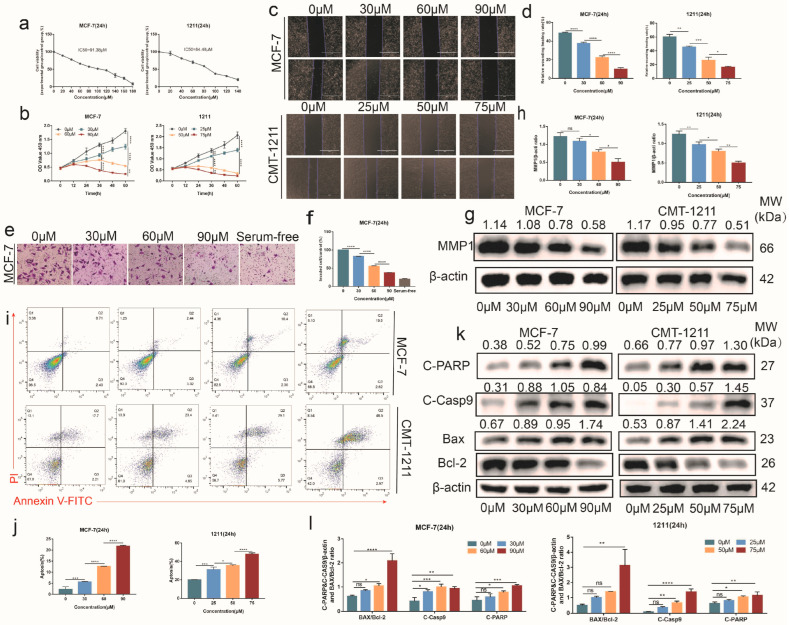
SNH inhibited the invasive and proliferative abilities of MCF-7 and CMT-1211 and promoted apoptosis. (**a**) Cell Counting Kit-8 kits were used to detect the activity of MCF-7 and CMT-1211 after treatment with different concentrations of SNH for 24 h. (**b**) Cell Counting Kit-8 kits were used to assess the cell activity of MCF-7 and CMT-1211 cells treated with different concentrations of SNH at 0, 12, 24, 36, 48, and 60 h. (**c**,**d**) Wound-healing assays were used to measure the cell migration capacity after SNH treatment for 24 h. Scale bar: 1000 μm. (**e**,**f**) Transwell chambers precoated with matrigel were used to examine the cell invasion ability of MCF-7 and CMT-1211 cells after SNH treatment for 24 h. Scale bar: 200 μm. (**g**,**h**) Western blot was used to detect the expression levels of MMP1 in MCF-7 and CMT-1211 cells treated with SNH for 24 h. (**i**,**j**) Flow cytometry was used to examine the effect of SNH on apoptosis. (**k**,**l**) Western blot was used to detect the expression levels of apoptosis-related proteins BAX, Bcl-2, cleaved caspase-9, and cleaved PARP in MCF-7 and CMT-1211 after being treated with SNH for 24 h. The western blot original images in the Figure S1. All results are expressed as mean ± SD of three independent experiments. * *p* < 0.05, ** *p* < 0.01, *** *p* < 0.001, **** *p* < 0.0001, ns = not significant.

Studies have shown that SNH exhibits a marked anti-proliferation effect on tumors. In order to explore the effect of SNH on breast cancer proliferation, MCF-7 and CMT-1211 cells were selected and treated with different concentrations of SNH. We found that SNH significantly decreased the cell viability of breast cancer cells, with a concentration-dependent decline. The IC50s of MCF-7 and CMT-1211 were 91.38 μM and 84.48 μM, respectively (Figure 3a). Compared to the control group, the MCF-7 mortality rate reached approximately 90 percent at a concentration of 180 μM, and the CMT-1211 mortality rate reached approximately 80 percent at a concentration of 140 μM (Figure 3a). In addition, compared with the control group, SNH had a more significant inhibitory effect on the proliferation of tumor cells as medication time increased (Figure 3b). The medicinal effects of administration over time were more recognizable.

Scratch assay and transwell assay were used to evaluate the migration and invasiveness ability of MCF-7 and CMT-1211 cells after treatment with SNH. The results showed that SNH could inhibit cell migration and invasiveness (Figure 3c–f). In addition, the Western blot results showed that the protein expression of MMP1 decreased as the concentration of SNH increased (Figure 3g,h). Additionally, apoptosis is an important process in maintaining the homeostasis of the cellular environment. The flow cytometry results showed that the apoptosis rate of SNH group was significantly higher than that of the control group through Annexin V/PI staining (Figure 3i,j). The Western blot results showed that the expression of cleaved caspase-9 and cleaved PARP and the ratio of BAX/Bcl-2 increased after SNH treatment (Figure 3k,l), which indicated apoptosis occurred in MCF-7 and CMT-1211. Combining the above results, we found that SNH could inhibit the migration capacity and invasiveness of breast cancer cells, promote cell apoptosis, and have conspicuous anti-tumor activity in vitro.

### 3.4. SNH Induced Cell Apoptosis by Increasing Intracellular ROS Level

**Figure 4:**
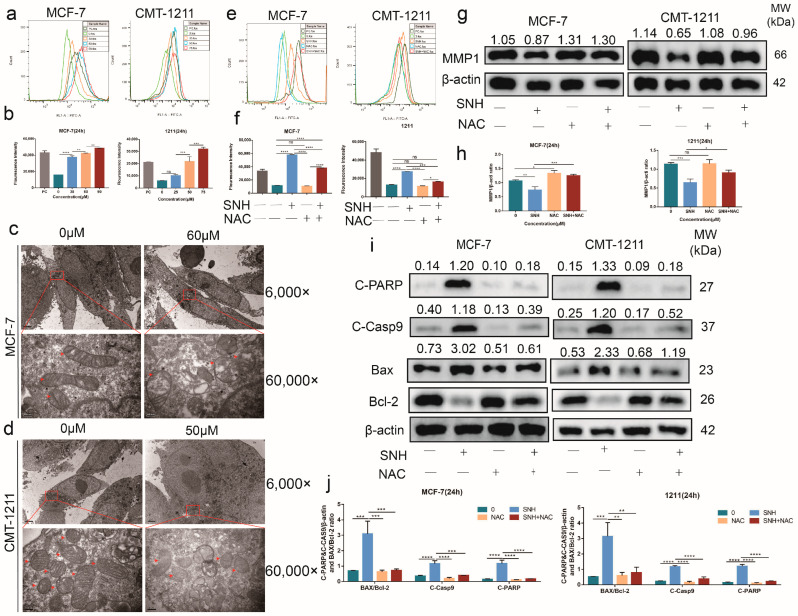
SNH induced apoptosis of MCF-7 and CMT-12111 by promoting the excessive accumulation of ROS. (**a**,**b**) Flow cytometry was used to detect the level of ROS in MCF-7 and CMT-1211 after treatment with different concentrations of SNH. (**c**,**d**) TEM was used to observe the mitochondria of MCF-7 and CMT-1211 after treatment with different concentrations of SNH. Red boxes and red arrows pointed to mitochondria. 6000× Bar: 2 μM; 60,000× Bar: 200 nm. (**e**,**f**) After treatment with 0 mM, NAC (10 mM), SNH (60 μM in MCF-7, 50 μM in CMT-1211), and NAC combined with SNH, flow cytometry was used to detect the level of ROS in MCF-7 and CMT-1211. (**g**,**h**) After treatment with 0 μM, NAC (10 mM), SNH (60 μM in MCF-7, 50 μM in CMT-1211), and NAC combined with SNH, the expression levels of MMP1 were analyzed using Western blot. The western blot original images in the Figure S1. (**i**,**j**) After treatment with 0 μM, NAC (10 mM), SNH (60 μM in MCF-7, 50 μM in CMT-1211), and NAC combined with SNH, Western blot was applied to analyze the expression levels of apoptosis-related proteins BAX, Bcl-2, cleaved caspase-9, and cleaved PARP in MCF-7 and CMT-1211. All results are expressed as mean ± SD of three independent experiments. * *p* < 0.05, ** *p* < 0.01, *** *p* < 0.001, **** *p* < 0.0001, ns = not significant.

An appropriate amount of ROS can promote the occurrence and development of cancer, but excessive production of ROS will show an anti-tumor effect. DCFH-DA probe was applied to detect the content of ROS in the cells. The flow cytometry results showed that, compared with the control group, the level of intracellular ROS in the SNH group was significantly higher (Figure 4a,b). Additionally, transmission electron microscopy images of MCF-7 and CMT-1211 revealed extensive damage to mitochondria after treatment with SNH, as follows: mitochondrial swelling, disrupted mitochondrial cristae (most disappeared), partial mitochondrial lysis, and heterogeneous mitochondrial matrix (Figure 4c,d). NAC is an ROS scavenger that can effectively reduce the generation of ROS. Compared with the SNH group, the ROS level of the combined NAC and SNH group was significantly lower (Figure 4e,f). At the same time, the Western blot results showed that the expression levels of MMP1 and Bcl-2 in the combined NAC and SNH group were partially restored, while the expression levels of BAX, cleaved caspase-9, and cleaved PARP decreased (Figure 4g–j). These results suggest that the overgeneration of ROS induced by SNH may be an important factor for apoptosis in MCF-7 and CMT-1211.

### 3.5. SNH Induced Apoptosis by Suppressing the Activation of the PDK1-AKT-GSK3β Pathway via ROS

**Figure 5:**
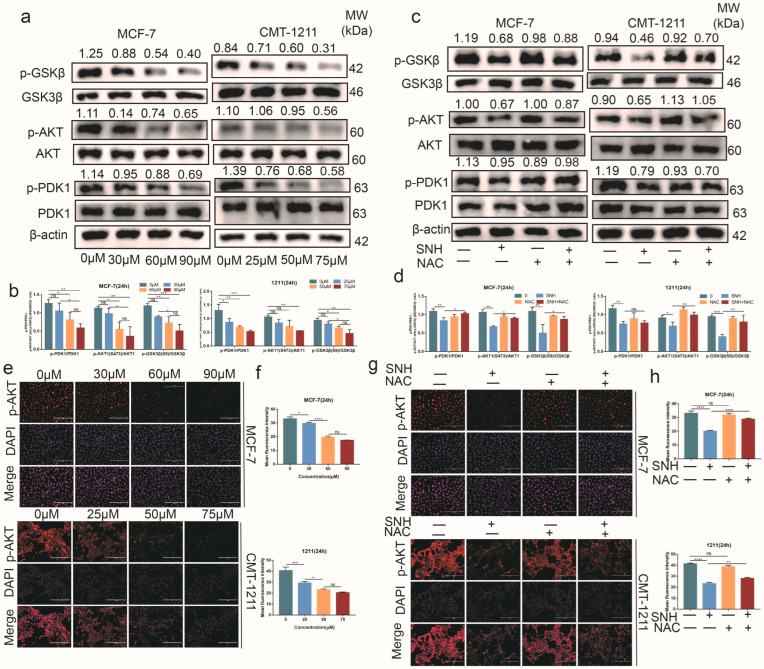
SNH acted by inhibiting the activation of the PDK1-AKT-GSK3β pathway. (**a**,**b**) After treatment with the different concentrations of SNH for 24 h, the expression levels of PDK1, p-PDK1, AKT, p-AKT (ser473), GSK3β, and p-GSK3β (ser9) were analyzed using Western blot. (**c**,**d**) After treatment with 0 μM, NAC (10 mM), SNH (60 μM in MCF-7, 50 μM in CMT-1211), and NAC combined with SNH, the expression levels of PDK1, p-PDK1, AKT, p-AKT (ser473), GSK3β, and p-GSK3β (ser9) were analyzed using Western blot. The western blot original images in the Figure S1. (**e**,**f**) After treatment with the different concentrations of SNH for 24 h, the expression level of p-AKT (ser473) was assessed using immunofluorescence staining. Scale bar: 200 μm. (**g**,**h**) After treatment with 0 μM, NAC (10 mM), SNH (60 μM in MCF-7, 50 μM in CMT-1211), and NAC combined with SNH, the expression level of p-AKT (ser473) was assessed using immunofluorescence staining. Scale bar: 200 μm. All results are expressed as mean ± SD of three independent experiments. * *p* < 0.05, ** *p* < 0.01, *** *p* < 0.001, **** *p* < 0.0001, ns = not significant.

At present, it is unclear whether SNH inhibits the activation of PI3K-AKT in MCF-7 and CMT-1211 cells through excessive production of ROS. The Western blot results showed that, compared with the control group, the phosphorylation levels of PDK1 and AKT were lower in the SNH group, as shown in Figure 5a,b. However, compared with SNH group, the expression levels of p-PDK1 and p-AKT were partially restored in the NAC and SNH combined group (Figure 5c,d), and immunofluorescence tests showed consistent results (Figure 5e–h). In addition, we detected the activity of GSK3β, a downstream target of AKT. GSK3β is a negative regulator in breast cancer. The results showed that as the SNH concentration increased, the expression level of GSK3β did not change significantly, but the phosphorylation level of GSK3β (ser9) showed a downward trend (Figure 5a,b). Correspondingly, the expression of p-GSK3β (ser9) was partially restored in the NAC and SNH combined group (Figure 5c,d). These results suggest that SNH could promote increased activity of the GSK3β protein. In conclusion, SNH inhibited the activation of PDK1-AKT-GSK3β by promoting the overgeneration of intracellular ROS.

### 3.6. SNH Inhibited the Growth of Breast Tumor 

**Figure 6:**
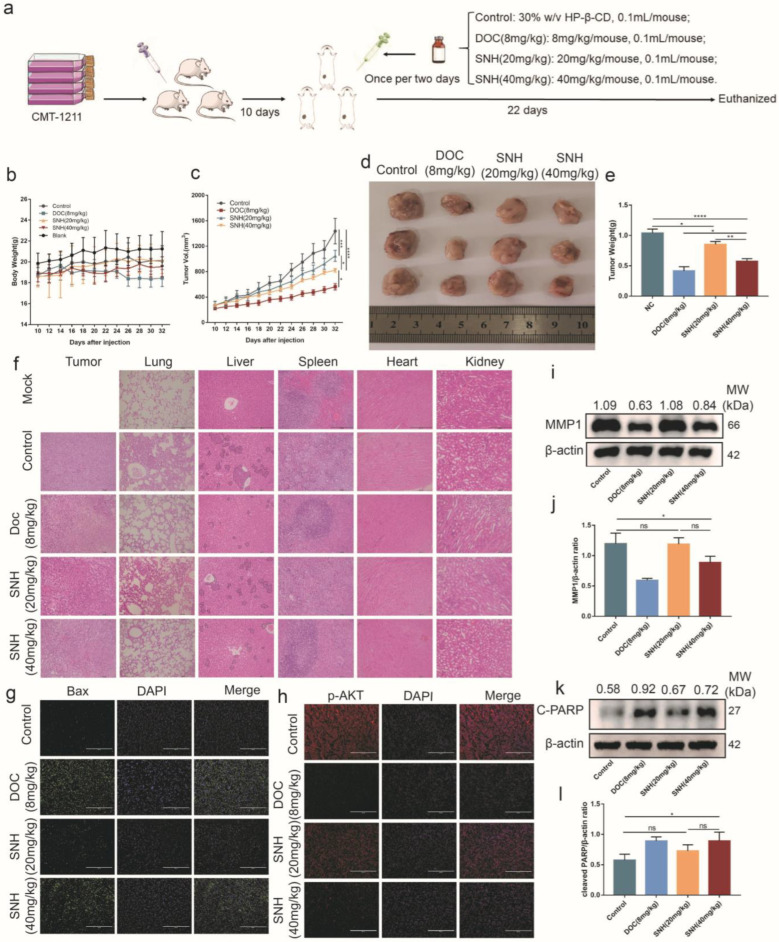
SNH was previously able to inhibit tumor growth in vivo. (**a**) Schematic diagram of establishing the mouse model of tumor and treatment regimen. (**b**) Changes in the body weight of mice in each group during treatment. (**c**) Changes in tumor volume during treatment in each group of mice. (**d**,**e**) The tumors of the mice were collected and weighed after the mice were euthanized. (**f**) H&E staining of mouse organs (lung, liver, spleen, heart, and kidney) and tumor tissues. (**g**,**h**) Immunofluorescence staining of p-AKT in tumors. Scale bar: 200 μm. (**i**,**j**) The expression of MMP1 in tumor tissues was analyzed using Western blot. (**k**,**l**) The expression of cleaved PARP in tumor tissues was analyzed using Western blot. The western blot original images in the Figure S1. All results are expressed as mean ± SD of three independent experiments. * *p* < 0.05, ** *p* < 0.01, *** *p* < 0.001, **** *p* < 0.0001, ns = not significant.

To further evaluate the effect of SNH on tumor growth in vivo, a subcutaneous homotransplant mouse model was established using CMT-1211 (Figure 6a). In order to better evaluate the effect of SNH, docetaxel (DOC) was added as a positive control group in the treatment of Balb/C mice. There was no significant difference in the changes in body weight among all treatment groups (Figure 6b). As shown in Figure 6c, treatment with DOC was the most effective among all the experimental groups, while the HP-β-CD group (control group, a solvent for SNH) had the fastest rate of tumor growth. Compared with the control group, both low-dose and high-dose SNH had significant inhibitory effects on breast tumors, and the therapeutic effect became more significant as the concentration increased (Figure 6c–e). The heart, spleen, and kidney pathological test results of each treatment group showed that there were no distinct abnormalities in the pathological sections. At the same time, histological testing showed that, in the control group, there were apparent tumor metastases in the liver (black line boxes) and lung, and the boundary between red pulp and white pulp in the spleen was obscure. In the treatment group, SNH resulted in elevated nuclear fragmentation of tumor tissue and reduced tumor metastases in the liver and lung as the SNH concentration increased (Figure 6f). 

In addition, the phosphorylation levels of p-AKT and BAX in tumor tissues were detected using an immunofluorescence assay. The results showed that, compared with the control group, the expression level of p-AKT was significantly lower in the SNH treatment group, while the expression level of BAX was significantly higher (Figure 6g,h). The expression levels of MMP1 and cleaved PARP were measured using Western blot. Similar to the in vitro results, SNH significantly inhibited the expression of MMP1 compared with the control group (Figure 6i,j). Cleaved PARP is one of the main indicators of apoptosis, and the expression level of cleaved PARP in the SNH treatment group was significantly higher than that in the control group (Figure 6k,l). Collectively, these results indicated that SNH has a significant anti-tumor effect in vivo. 

## 4. Discussion

It has been reported that gene mutations (breast cancer genes BRCA 1 and BRCA 2, etc. [25]) can result in the emergence of breast cancer and promote the survival and metastasis of cancer cells [26]. Through statistical analysis of the GEO database, we found certain breast cancer-related DEGs that had a strong relationship with the invasiveness and apoptosis of breast cancer and cancer-related pathways. Breast cancer is one of the tumors with a high mortality rate, and its heterogeneity and drug resistance make the clinical treatment of breast cancer very challenging [27]. However, the active ingredients in traditional Chinese medicines and their derivatives have attracted people’s attention because of their great therapeutic potential. Therefore, it is meaningful to find active ingredients of traditional Chinese medicine that can treat breast cancer effectively.

SNH, a derivative of houttuynium, has been used in the clinical treatment of pelvic inflammatory disease. SNH has also been found to have anti-tumor activity [9,10]. Nevertheless, few studies have investigated the influence of SNH on breast cancer. In this study, a mouse mammary tumor model was successfully established, and SNH was found to exhibit significant anti-breast cancer activity both in vivo and in vitro. We found that SNH could induce the overgeneration of ROS and result in mitochondrial dysfunction in MCF-7 and CMT-1211 cells. The overgeneration of ROS inactivated the PI3K-AKT pathway, thereby increasing GSK3β activity, increasing the expression level of BAX, cleaved caspase-9, and cleaved PARP, and finally causing DNA damage in MCF-7 and CMT-1211 cells.

Previous studies have shown that SNH has an important effect on the proliferation and invasiveness of non-small cell lung cancer [10]. In light of the similarity between canine breast cancer and human breast cancer [28] and the tumor-forming ability of canine mammary tumor cell CMT-1211 in Balb/C mice [29], two cell lines (CMT-1211 and MCF-7) were selected. The results showed that SNH could significantly inhibit the growth of breast tumors in vivo. Meanwhile, we also found that SNH inhibited the proliferation and migration of breast cancer cells in vitro, as evidenced by the wound healing rates. In addition, the flow cytometry results showed that the apoptosis rates of MCF-7 and CMT-1211 increased, which was also demonstrated using Western blot analysis. All the results aligned with previous reports [10,30]. When cells receive apoptotic signals, BAX and Bcl-2 are recruited to the outer mitochondrial membrane to interact and activate, which will induce mitochondrial damage [31,32,33]. In this research, it was found that the ratio of BAX/Bcl-2 increased. This suggests that the pro-apoptotic effect of SNH may be relevant to mitochondrial function.

As a commonly used anti-inflammatory drug in clinical practice, SNH has been proven to exert anti-inflammatory effects by increasing the level of ROS [6]. Studies have shown that ROS has a close relationship with cancer and that the excessive accumulation of ROS can cause mitochondrial membrane potential reduction, thereby inducing mitochondrial dysfunction, bioenergy failure, and apoptosis [13,34,35]. In our study, we found that SNH could promote intracellular ROS overgeneration using flow cytometry, which enhanced along with the increase in SNH concentration, and this phenomenon was partially inhibited by NAC. In addition, the Western blot results showed that compared with the SNH group, the expressions of BAX, cleaved caspase-9, and cleaved PARP in MCF-7 and CMT-1211 cells were lower in the combined NAC and SNH group, and the expression of Bcl-2 was higher than that in SNH group. These results suggest that SNH may cause mitochondrial dysfunction and initiate cell apoptosis by promoting the production of ROS.

As a by-product of cell metabolism, ROS participates in the regulation of the PI3K-AKT pathway [36]. Low or moderate levels of ROS can activate the PI3K-AKT pathway and inhibit apoptosis [37], while excessive ROS can be a negative regulator of this pathway [38,39]. As a direct downstream target of AKT, GSK3β acts as a tumor suppressor in breast cancer, mediating the expression of cyclin D1 to regulate the cell cycle and increase chemosensitivity [40,41]. Phosphorylation of serine 9 of GSK3β reduces the activity of GSK3β, while phosphorylation of tyrosine 216 of GSK3β enhances the activity of GSK3β [21]. GSK3β can be present in the cytoplasm and nucleus, but the activity of nuclear GSK3β is higher than that of cytosolic GSK3β. In the process of apoptosis signaling, nuclear GSK3β regulates a large number of transcription factors and encodes apoptotic regulatory proteins that target mitochondria. For example, GSK3β can form a complex with p53 to induce the expression of BAX [42]. The polymerized BAX outside of mitochondria facilitates the release of apoptotic proteins from mitochondria into the cytoplasm, where these proteins (such as cytochrome c, apoptotic protease activating factor-1, ATP/dATP, and caspase-9) assemble apoptotic bodies, triggering the caspase cascade [43]. In this study, the Western blot results showed that the expressions of p-PDK1, p-AKT1, and p-GSK3β (ser9) decreased, which represented a concentration gradient-dependent pattern, suggesting that SNH could inactivate the PI3K-AKT pathway, increase the activity of the GSK3β protein, and inhibit the cell viability of MCF-7 and CMT-1211. Abnormal activation of the PI3K pathway is one of the most common phenomena in the development of breast cancer. Therefore, inhibitors targeting the PI3K signaling pathway are promising drugs for treating breast cancer. Currently, there are several drugs that target the PI3K pathway: pan-PI3K inhibitor, PI3K isoform-specific inhibitors, AKT inhibitor, rapamycin analogue or mTOR inhibitors, etc. [44]. Studies have shown that PI3K-AKT signaling activates estrogen receptor α in an estrogen-independent manner, and AKT overexpression protects breast cancer cells from tamoxifen-induced apoptosis [45]. This indicates that inhibition of PI3K can enhance the therapeutic effect on ER+ breast cancer cells. In addition, some drugs developed according to the molecular structure and function of natural products have been shown to be able to treat breast cancer by inhibiting the overactivation of the PI3K pathway. For example, it has been proven that quercetin suppressed the activation of PI3K and AKT, which increased the ratio of BAX/Bcl-2 to induce apoptosis of breast cancer cells, as well as significantly inhibiting the growth and metastasis of CD44+/CD24 breast cancer stem cells in vivo [46]. In a study of ginsenoside treating MDA-MB-231 cells and HUVEC cells, it was found that ginsenoside could reduce intracellular AKT/mTOR/p70S6K and hypoxia inducible factor-1-α. The activation of vascular endothelial growth factor receptor 2 in HUVECs induced by vascular endothelial growth factor was eliminated [47]. In this study, it was found that SNH induced mitochondrial dysfunction by promoting excessive generation of ROS and inactivated the PDK1-AKT signaling pathway, which activated GSK3β activity. Activated GSK3β entered the nucleus, and regulated the expression of BAX in the nucleus and polymerization of BAX in the mitochondrial outer membrane, triggering the mitochondrial apoptotic pathway, including an increased BAX/Bcl-2 ratio and caspase cascade. On the basis of the above-mentioned results, it is suggested that SNH potentially has a therapeutic effect on breast cancer. 

## 5. Conclusions

In conclusion, the occurrence of breast cancer is involved in a large number of DEGs that participate in cancer-related pathways. SNH can promote the excessive accumulation of ROS in MCF-7 and CMT-1211 cells, target the PI3K-AKT-GSK3β pathway to induce cell apoptosis in vitro, and significantly inhibit the growth of breast tumors in vivo. Thus, SNH may be a potential drug for breast cancer treatment.

## Data Availability

The datasets generated and analyzed during this study are available from the corresponding author on reasonable request. All data and materials, as well as software applications, support their published claims and comply with field standards.

## Supplementary Materials

The following supporting information can be downloaded at: https://www.mdpi.com/article/10.3390/cancers15051614/s1, Figure S1: The western blot original images.

## Abbreviations

AKT	Protein kinase B
BAX	Bcl2-Associated X
Bcl-2	B-cell lymphoma-2
Casp9	Caspase 9
CCK-8	Cell Counting Kit-8
DCFH-DA	2’,7’-Dichlorodihydrofluorescein diacetate
GO	Gene Ontology
GSK3β	Glycogensynthasekinase-3β
KEGG	Kyoto Encyclopedia of Genes and Genomes
NAC	N-Acetyl-L-cysteine
PARP	Poly ADP-ribose polymerase
PDK1	3-phosphoinositide-dependent protein kinase-1
PI3K	Phosphatidylinositol3-kinase
PMSF	Phenylmethanesulfonyl fluoride
RIPA	Radio Immunoprecipitation Assay
ROS	Reactive oxygen species
SNH	Sodium new houttuyfonate
